# FUNCTIONAL AGING TRAJECTORIES AND DRUG INTERACTIONS

**DOI:** 10.1093/geroni/igac059.965

**Published:** 2022-12-20

**Authors:** Thais Lopes Deliveira, Sara Hägg

**Affiliations:** Karolinska Institutet, Stockholm, Sodermanlands Lan, Sweden; Karolinska Institutet, Stockholm, Stockholms Lan, Sweden

## Abstract

Markers of functional aging can be used to track biological aging longitudinally and how it changes in response to the environment, e.g., drugs. We aimed to investigate how different drug classes may alter functional aging trajectories using data from the National E-infrastructure of Aging Research (NEAR) in Sweden. Data were harmonized across several longitudinal cohorts of aging for general cognitive performance, grip strength, walking speed, sensory ability (visual and hearing), lung function and assessment of frailty using the accumulation deficit model known as the frailty index. Selected drug classes were lipid lowering, glucose lowering and blood pressure lowering medications that are commonly used in old adults. Preliminary analysis using data from one longitudinal cohort shows that using glucose lowering drugs was associated with lower frailty. Additional analyses are ongoing to increase sample sizes. We anticipate that several drug classes may be important for changing functional aging trajectories in late life.

